# A Remarkable Verification

**Published:** 1887-11

**Authors:** 


// HALL'S
### JOURNAL OF HEALTH

TRUTH DEMANDS NO SACRIFICE; ERROR CAN MAKE NONE.

VOL. 34. NOVEMBER, 1887. No. 11.

### SPECIAL.

We desire to call attention to the newly advertised list of premiums to *individuals* and *clubs* remitting their subscriptions directly to this office. There is no sham about it, as any person at all conversant with HALL'S JOURNAL OF HEALTH, now in its thirty-fourth year, will be able to testify. Let the good work go on. We mean to send out a greater number than any similar publication in this or any other country. Who will be first?

### A REMARKABLE VERIFICATION.

The readers of the JOURNAL will call to mind, that in its May number, we published a reduced electro-photograph of a life size crayon drawing, which was given out to be the likeness of an Egyptian Astronomer etc., of the seventh century, who has on various occasions, signified his presence and personality to Mrs. Harriet E. Beach of New York City, towards whom he appears to be drawn as a spiritual friend, philosopher and guide. We then told the story of the production of the crayon through the instrumentality of those two remarkable sensitives, Doctor and Mrs. Henry Rogers aided by the presence of Mrs. Beach, and personally vouched for its truth, as we felt authorized to do, from similar experiences had with these two instruments, and particularly one when two such portraits were taken by no mortal hand, one life size, fully recognized by the whilom husband, and the other of cabinet size, being that of an occultist in the days of long ago, an account of which was published in the *Boston Banner of Light.*

Now then for a most remarkable verification of the presence and personality of that denizen of the world of spirits, who has in a manner attached himself to Mrs. Beach, giving his name as Amarona. It was on the 15th day of last July, that Mrs. Beach, then a stranger to the sensitive Mr. W. M. Keeler, who resides at 1227 Bedford avenue, Brooklyn, applied to him for a sitting for what is termed spirit photography. In order that the result, if successful, might be more satisfactory to her mind, Mrs. Beach did not disclose her identity, a thing of no consequence to the sensitive. Under these circumstances the sitting was had, and quite recently Mrs. Beach called at the Journal office, and presented us with a copy of the photograph thus obtained. In the centre of the picture is a distinct three quarters likeness of Mrs. Beach, seated in a chair, and to her left, covering about one third of the plate, is a standing male figure, the lower folds of whose surplice-like garment, are curiously interblended with her own, from her left shoulder downward.

This male figure in form, feature, expression, pose and apparel, is unmistakably the same as that of the crayon produced through the instrumentality of the Rogers's, although lacking in some of the frontal ensignia, and being of greater relative length. It will be remembered that the crayon was of life size, whilst the photograph measures across the eyebrows, in a straight line between the turban folds, only seven sixteenths of an inch. But this is not all; after this photograph was taken as above stated, Mr Keeler invited his sitter to enter an adjoining room for an experiment in slate writing by the unseen powers.

Upon being seated there, Mrs. Beach states that she wrote questions addressed to several names on small slips of paper, and placed them between a pair of slates, which she bound together with her pocket handkerchief, holding the slates meanwhile with her two hands, the sensitive Keeler, placing one hand upon them. Writing was then heard inside the slates, and upon separating the two, the under slate was found written completely over with messages in different languages, including one in Egyptian, which being deciphered, reads as follows: "Dear Charge, This is only the beginning of what is to come. Such grand results are only to be achieved through you. This is a happy occasion, Amarona."

Mrs. Beach has had these slate writings photographed, and has offered them to be photo-lithographed and published with her statement of the manner of their production in detail, and it is probable that they will soon be given to the public in that form. In this relation it is proper to state that Mrs. Beach is a woman of culture and refinement, whose interest in the occult science, has led her into close relations with forces and intelligences, far better understood in remote ages than they are to-day, and it is largely through her own powers as a sensitive, that the phenomena of which we speak, are able to be manifested.

To recapitulate; for some time prior to January last, Mrs. Harriet E.

Beach of New York City, had held frequent communication with an invisible intelligence, through the instrumentality of a well-known sensitive, by means of whose intermedial agency he was also able on occasion, to present himself in visible form.

On one or more occasions this supermundane presence informed Mrs. Beach, that should she desire it, he would be able to give her his likeness, through the agency of Dr. and Mrs. Henry Rogers, sensitives for this phase of manifestation, then located near her residence. Thereupon Mrs. Beach made arrangements with Dr. Rogers for a series of sittings, having in view the promised result. On January 31st, the final sitting was had, the outcome being a life size head and bust crayon drawing of spirit Amarona, wrought in darkness by no visible hand, an electrotype of which appeared on the second page of our May issue. On July 15th, following, Mrs. Beach as a stranger, applied to Mr. Keeler of Brooklyn, N. Y., a photographic sensitive, for a sitting with him. This was permitted, and on the same plate with her likeness appeared in full view a likeness in miniature, the exact counterpart of the life size crayon previously obtained, as above narrated. Indeed the most careless observer would recognize them as being one and the same. Then followed the confirmatory message of spirit Amarona, by independent slate writing, under circumstances which preclude the suspicion of collusion or fraud. Here is a chain of circumstances which ought to satisfy even the more skeptically minded of our readers. They do not oppose themselves to any form of religious sentiment worthy of the name. On the contrary, they go far towards proving as true, that which forms the basis of every known religion : the continuance of conscious individual existence of mortals in the great hereafter. Why then the bitter opposition to those sublime efforts of the spirit world, to roll aside the clouds of ignorance, which have so long obscured the light which makes clear the way of life here, and the certainty of its unbroken progression in the world to come ?

Is it out of fear that the lame and tottering myths of pagan superstition, will be called upon to give place to a more reasonable conception of the Divine purpose ? They have already lost their force as terrorizing factors, and many a "perturbed mind" that kept the body sick with thoughts of the coming "judgment," wherein vengence was ever uppermost, has grown calm under the more healthful aspect of love, mercy and redemption. The allegory of the creation, the story of Adam and Eve, of original sin and possible redemption, are no longer held up as bugbears to enlightened congregations. The age has outgrown it. Patient and scholarly researches into the obscurity of the past, have revealed their mythical origin, and the religion of the future will walk the earth as it walks the heavens, the companion of love and the handmaid of science.

